# Heart Health Risk Assessment System: A Nonintrusive Proposal Using Ontologies and Expert Rules

**DOI:** 10.1155/2014/959645

**Published:** 2014-06-15

**Authors:** Teresa Garcia-Valverde, Andrés Muñoz, Francisco Arcas, Andrés Bueno-Crespo, Alberto Caballero

**Affiliations:** Departamento de Ingeniería Informática, Universidad Católica San Antonio, Guadalupe, 30107 Murcia, Spain

## Abstract

According to the World Health Organization, the world's leading cause of death is heart disease, with nearly two million deaths per year. Although some factors are not possible to change, there are some keys that help to prevent heart diseases. One of the most important keys is to keep an active daily life, with moderate exercise. However, deciding what a moderate exercise is or when a slightly abnormal heart rate value is a risk depends on the person and the activity. In this paper we propose a context-aware system that is able to determine the activity the person is performing in an unobtrusive way. Then, we have defined ontology to represent the available knowledge about the person (biometric data, fitness status, medical information, etc.) and her current activity (level of intensity, heart rate recommended for that activity, etc.). With such knowledge, a set of expert rules based on this ontology are involved in a reasoning process to infer levels of alerts or suggestions for the users when the intensity of the activity is detected as dangerous for her health. We show how this approach can be accomplished by using only everyday devices such as a smartphone and a smartwatch.

## 1. Introduction

Heart diseases are one of the most frequent causes of mortality and responsible for nearly two million deaths a year. In many cases significantly pathological tests in hospital can be used as a basis for detecting patients at risk for heart disease. However, one of the problems of disease prevention involves deciding when a slightly abnormal value is a risk and when a normal value can be a risk for a specific person according to her features (e.g., age, weight, etc.) and/or her current physical activity. Besides, when people are not confined to hospitals, such information is not available and a medical problem may not be detected in time.

In that sense, computational techniques are acquiring a high importance in order to develop flexible and accurate models of complex biological systems, specifically in the health domain. Indeed, in recent years, rapid developments in hardware and built-in sensors have generated the possibility of sensing a large volume of biometric data. Specifically, it is growing the use of data mining techniques to solve biological problems by analyzing large biological datasets [1].

Modeling knowledge by means of ontologies in the medical domain, especially in the e-health area, is an active research field. In bioinformatics, ontology-based systems provide reusable terminology resources and they can be used to improve the management of complex systems [2–4]. Thanks to these ontologies, different context information (e.g., biometric data, user's activity and medical information, etc.) can be captured and validated. Furthermore, this terminology enables the definition of expert rules, which in turn are the input to reasoning processes aimed to infer new knowledge. Thus, the adoption of ontologies in our approach is oriented to empower the inference of alert situations from the user's current context.

In this work the heart rate and the intensity of the physical activity are used to detect abnormal situations during the execution of such activities. The approach takes into account the variability between different people (age, health status, fitness level, etc.). All this information is modeled through ontology and a set of expert rules that form a context-aware system designed to provide a personalized response when an unexpected heart rate is detected. Here we introduce all these ideas in a research proposal format in order to provide the guidelines and technical details of our system. Therefore, a complete evaluation of our system cannot be supplied at this point and instead we present a thorough study of the different parts that compose it.

The main contributions in this paper are twofold. First, the physical activity is determined using data mining techniques over data gathered from nonintrusive wearable sensors. Second, the definition of SHCOntology (simple health care ontology) allows us to represent a health care context in order to express expert rules. Such rules capture alert situations according to the activity that the person is currently performing, her heart rate, age, fitness status, and other conditions, such as if the person suffers some cardiac dysfunction.

The rest of the paper is structured as follows.  Section 2 defines the assessment system architecture. Next, Section 3 introduces the proposed sensors deployment and data mining techniques used to determine users' activities. Empirical results on the viability of our approach are showed in this section. Then, Section 4 sets the theoretical base about the importance of overreaching and overtraining related to some heart health problems.  Section 5 presents the knowledge model used to assess heart health risks according to the person's context. Later, Section 6 shows how this approach is used in two specific scenarios.  Section 7 discusses some related works. Finally, conclusion and future work are given in Section 8.

## 2. Assessment System Architecture

The rapid growth in hardware technology has become an attractive option to collect data using a wide set of available sensors. However, there is a need to rely on nonintrusive sensors trying to keep a minimal deployment and especially to avoid annoying users. Indeed, built-in cheap sensors are now integrated into daily use devices, such as mobile phones. Exploiting built-in sensors leads us to the possibility of large-scale human sensing and collecting large datasets [5].

Under such conditions, we propose the use of inertial sensors from a smartphone and a smartwatch to predict the individual's activity. As a result, we can use this knowledge to provide her with some important information about her health status.

**Figure 1:**
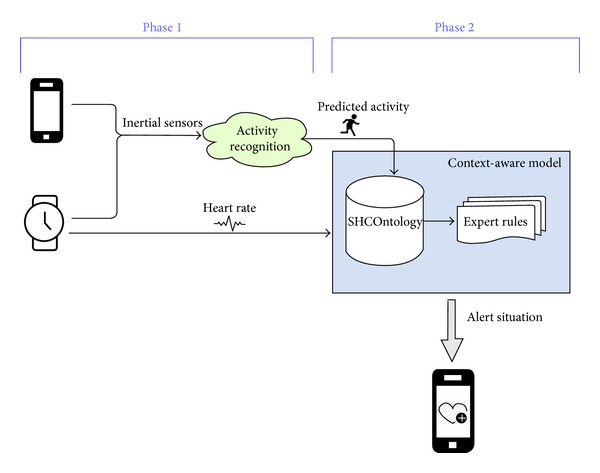
Overall architecture.

With the aim of extracting such relevant information, this paper proposes a system which involves two different phases.  Figure 1 shows the overall architecture of the system.

The first phase involves a physical activity recognition using data mining techniques over the data gathered from inertial sensors in a smartphone and a smartwatch (see Section 3). The use of data mining over a data intensive framework provides the possibility of detecting and learning behaviors and patterns from biological domain problems.

In fact, bioinformatics research entails many problems that can be solved by data mining tasks. Concretely, physical activity recognition using wearable sensors can provide valuable information regarding individual's movements and help us to determine some aspects of her health. Another valuable information about the individual's health is her heart rate. In this first phase of our proposal, this information is also monitored and gathered directly from the heart rate sensors in the smartwatch. Note that, in this phase, the user configures her smartphone with her own features (age, weight, height,…) in order to personalize the system. These parameters should be configured only the first time that the system is used.

Once the activity has been determined and the heart rate collected, this information is forwarded to the context-aware model, starting the second phase of our proposal (see Section 5). The context-aware model is responsible for representing and reasoning over this information in order to detect possible alert situations. The reasoning model is a compound of the SCHOntology and a set of expert rules. The SCHOntology represents our health care domain and comprehends various aspects of the users and their activities, including biometric data, possible diseases, fitness status, and intensity of the activity. Based on the SHCOntology, a set of expert rules are defined to infer alert situations and suggestions according to the available knowledge about the person.

Observe that the first phase of our system is data-driven; that is, we construct a classifying model from previous data. On the other hand, the second phase is knowledge-driven; that is to say, models are now created by experts. By combining both approaches, the goal of our two-phase system is to offer an app for early detection of overreaching of a person while she is doing her normal life or performing some exercise.

Finally, it is worth mentioning that the app will include a disclaimer in order to inform the user that it is intended for informational purposes only and should not be used as a medical diagnostic device. It should be noted that the system does not purport to decide about a medical relevant condition of the users. It is limited to offer a set of recommendations about several alert situations based on experts' knowledge while using everyday devices, without the need of obtrusive and annoying sensors.

## 3. Activity Recognition

The following sections explain the activity recognition phase of our proposal. Specifically, first subsection describes the components needed to gather the relevant data from a person when she is performing everyday activity and how the inherent noise and imprecision of the data are removed.

Second subsection shows how data mining techniques are able to determine the activity from the inertial sensors data. This approach is validated over real data from the PAMAP2 physical activity monitoring dataset [6].

### 3.1. Sensor Devices and Data Collection

In this work sensors signals from a smartphone and a multisensor wristband (smartwatch) are used as the base signal to classify the physical activity that the subject is performing. Concretely, accelerometers, gyroscope, and magnetoresistive (AMR) magnetic sensors are proposed to determine the activity.

Smartwatches are wearable devices that use sensors to gather data about the user's movement and other features. First examples of wristband sensors were based on the use of accelerometers to measure steps and other movements [7]. However, the latest models already include other kinds of metrics like heart rate monitoring, temperature, or blood oxygen levels using infrared sensor [8].

Regarding smartphones, almost all the last models available on the market include a growing set of cheap powerful embedded inertial sensors [9]. More specifically, triaxial accelerometers, gyroscopes, and magnetometers are commonly found on smartphones and tablets. Under these conditions, our approach can take advantage of these inertial sensors by only requiring that the users wear the smartphone in a pocket near her chest.

Therefore, activity data could be collected from the aforementioned sensors in a noninvasive way. However, the use of real time data from inertial sensors is subject to noise and imprecision [10]. As a result, there is a need to filter out noise before using the data for activity recognition. In order to address this issue we propose a moving average filter of order 3. This simple method has been shown to be able to remove the random noise [11].

Finally, to make our system activity-aware requires a feature processing and a classification method able to infer what activity an individual is engaged in. In that sense, this work proposes the use of data mining techniques in order to predict users' activities throughout the day. Data mining techniques provide the ability of analyzing large datasets to infer patterns and generalizations. Then, predictive models can be built using classification algorithms which are used to predict the physical activity of the individual.

### 3.2. Feature Processing and Prediction Model

As described in Section 3.1, data mining techniques enable inferring patterns and generalizations from large datasets. It can be used to build models that allow classifying between several predefined classes (see [12] for further explanation on this topic). In this work the use of data mining techniques is aimed at analyzing raw data from sensors in order to determine what physical activity the user is performing.

With the aim of validating the described approach, a dataset for the analysis and classification of physical activity was obtained from PAMAP2, as mentioned above. It contains data from 9 subjects performing 18 different physical activities with different intensities (to ascend/descend stairs, to be seated, ironing,…). Therefore, we use the same classification of activities as in the PAMAP2 dataset. A brief description of each of these activities can be found attached to the published dataset.

Subjects wear 3 inertial measurement units (IMU) and a heart rate monitor. Each IMU contains two 3-axis accelerometers, a 3-axis gyroscope, and a 3-axis magnetoresistive (AMR) magnetic sensor. The sensors are placed onto 3 different body positions: chest, wrist, and ankle. The heart rate monitor is attached to a chest strap.

This dataset provides a way to simulate the real data from sensors. Consequently, it is possible to validate the feasibility of the approach through this simulation. Furthermore, it provides an easy method to evaluate different algorithms in order to determine the accuracy of each technique and select the most appropriated one.

Different classification methods using WEKA are successfully used over the data in order to build a model to determine the specific physical activity [6]. Authors have shown that, given the IMUs and heart rate values for a person in an instant *t*, the model is able to recognize the physical activity with a high accuracy (over 90% of success).

However, in systems for physical activity monitoring, the number of sensor placements should be kept as low as possible for reasons of practicability and comfort [6]. In fact, our systems should be used during all daily activities in order to provide a quick heart health risk assessment. Therefore, comfortable, easy to handle, and wearable devices should be used.

Since the PAMAP2 study allows us to determine the physical activity using all the dataset with a 90% of success, in this work we have investigated whether it is possible to determine the activity without the use of some data from the original dataset. With this aim, we preprocess the data removing different variables in turn (e.g., heart rate, chest sensors, wrist sensors,…).

After several tests, it has been shown that the resulting classification models are able to determine the activity just using the inertial chest and wrist sensors. Note that this fact does not imply that there are no additional correlations between the rest of variables and the activity. The tests also show that the algorithms classify the activity with a high accuracy using a reduced dataset.

Table 1 shows the results from the tests performed with the original dataset (i.e., using all variables) and the results using the reduced tests (i.e., using just the wrist and chest sensors). Likewise, Table 1 shows the best results obtained from the different algorithms for the full and reduced datasets: boosted C4.5 decision tree (confidenceFactor = 0.15, minNumObj = 50) and kNN (kNN = 7, *W* = 0).

Observe that although the precision and the accuracy are lower in the tests with the reduced dataset, they are still very high. As a result, it is affordable to reduce the number of variables in order to get a less intrusive system.

## 4. Biomechanics of Cardiopulmonary Effort

This section deals with the theoretical background in biomechanics as the starting point to design our knowledge model and expert rules.  Section 4.1 reveals the importance of the workload in aerobic or anaerobic exercises with respect to the person's physiology, fitness status, and health features.  Section 4.2 deals with the estimation of quantifiable units of effort such as metabolic equivalent of task (MET) and relative maximal heart rate intensity in order to use such measures in expert rules.

### 4.1. Activity Load

Overreaching and overtraining are the accumulation of training over one's capacity, which may result in a short-term decrease in performance capacity and maybe in a serious heart problem. In high-performance athletes, the overreaching is common and intentionally induced as part of a training regimen. However, in popular athletes, specially beginners and people with critical problems, it could be a frontier to avoid trespassing [13]. There are several risk factors like highly motivated athletes who respond to poor athletic performance by increasing training loads, athletes without individualized training who think that they do not get enough exercise and, more worrying, people without a good physical shape or with some disease [14].

The general prevention about exercise overreaching—especially in persons with some disease—must be based on a test set like blood testing or coordination study that cannot be self-made. Fortunately, there are other prevention alternatives such as daily training log or activity monitoring based on accelerometers and heart rate sensors that can be performed daily without control of a specialist, as explained in Section 3.

The activity load and his effort level determine the cumulative strain involved with training. To be useful in early detection of overreaching, it is necessary that a systematic documentation of subjective and objective factors must be completed at baseline and reevaluated regularly. The daily/weekly variations that occurred in the training log effort of a person can help to identify an individual athlete's abnormal response to training at an early stage. This baseline must be combined with the health level of a person and her possible diseases. Once identified, interventions can be made to prevent further deterioration and normalization of subjective and objective criteria.

A workout log includes intensity, duration, and mode of training based on accelerometer sensors along with a rating of perceived exertion (RPE) for the entire training session on a specific day, for example, with the Borg scale [15]. This scale rates perceived effort from 6 (20% of effort) to 20 (exhaustion). The RPE session can be recorded and represents an objective measurement of daily raining load [16]. Actually, the subjective perception is combined with other objective indexes of physical activity, such as pulse or effort (i.e., power obtained from sensor accelerometers).

### 4.2. MET and Relative Maximal Heart Rate Intensity Estimation

We need to express the activity intensity in a quantifiable unit that could be used in expert rules. With this aim, the term metabolic equivalent (MET), a well-known measure in this area, is one of the most adopted alternatives. The basic MET unit is equal to VO_2_ (oxygen consumption) in a resting state, equivalent to 3.5 ml · kg^−1^ · min^−1^ [17]. The most common technique used to measure oxygen consumption nowadays is the open-circuit spirometry that depends on complex laboratory equipment managed by skilled people. However, there have been several reports about how to calculate the VO_2max⁡_ in function of parameters such as gender, country, ethnicity, activity level, body weight, and height of the subject [18]. Most of these studies concluded that the prediction of VO_2max⁡_ must be based on age and gender. This VO_2max⁡_ factor is relative to the duration and intensity of the effort that depends on the training session estimated by the RPE.

The most comprehensive equations for calculating VO_2max⁡_ are shown in (1) (males) and (2) (females) [19]

(1) VO2max⁡  (males)  =((0.072·Ht)−0.052)   ·(44.220−(0.390·Age))+(0.006·Wt),

(2) VO2max⁡  (females) =((0.063·Ht)−0.045)  ·(37.030−(0.370·Age))+(0.006·Wt),

where Ht represents the person's height measured in meters, Wt is her weight in kilograms, and Age represents her age expressed in years.

As described above, measuring oxygen consumption implies an open-circuit spirometer in a specific laboratory. However, such method does not provide users to know their VO_2_ levels while they are doing their normal activities. Because of that, the most popular, simple, and practical method to estimate the current intensity in VO_2_ of a sport activity is done by the heart rate (HR). It allows us a nonintrusive manner for measuring the oxygen consumption by a conventional HR monitor.

The HR is one of the most important variables to measure the intensity in the physical activity. The minimal intensity threshold is 55% to 65% of maximal heart rate (HR_max⁡_) [20]. This range is based on individual variability in the exercise intensity necessary to improve the aerobic fitness. Those with low aerobic fitness will achieve fitness improvements by training at a lower level of this range. Competitive athletes will need to train at higher intensities than people just interested in improving her health. The highest level of the range is approximately at 94% of HR_max⁡_. Nevertheless, most people get the optimal values of intensity between the 77% and 90% of their HR_max⁡_ [17].

Since there is a relatively linear relationship between HR and exercise intensity, it is possible to use (3), where the current VO_2_ is obtained as a linear regression formula from experimental data gathered by Swain et al. [21]
(3)
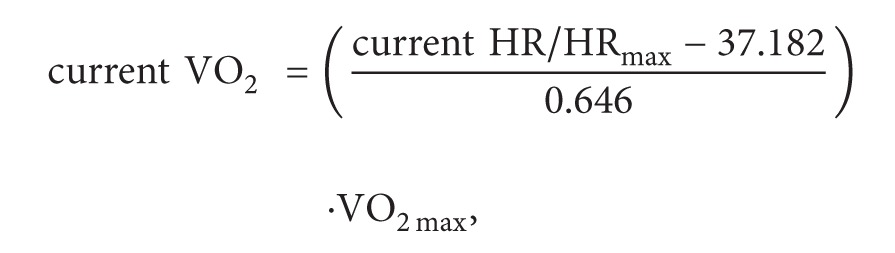

where the VO_2max⁡_ is calculated by (1), (2) and the HR_max⁡_ can be estimated by several ways.

Several formulas are used to estimate individual maximum heart rates, mostly based on age. For example, the simple Haskell's formula [22], (4), Tanaka's formula [23], (5), or the more recent Gellish's formula [24], (6), for people from 30 to 75 years with a standard deviation of 6 to 15 bpm according to the equation. As a result, with simple data such as gender, age, weight, height, and current HR we can obtain derived data like HR_max⁡_, VO_2max⁡_, and current  VO_2_.  Table 2 shows some equivalence between the described parameters and the mentioned formulas are given next

(4)  Haskell:  HRmax⁡=220−age   variation  of  ±15 bpm,

(5)  Tanaka:  HRmax⁡=208−0.7·age,

(6)  Gellis:  HRmax⁡=207−0.7·age   variation  of  6  to  15 bpm.

The most used equation to estimate the HR_max⁡_ is Haskell's formula [25]. This equation indicates that the HR_max⁡_ decreases approximately from the 5% to 7% per decade. However, actually, the HR_max⁡_ decreases from the 3% to 5% in such a period. Therefore, the equation results in error when the HR_max⁡_ is estimated for people over 40 years old approximately. Indeed, there are some works describing this fact [23, 26]. According to these works, Haskell's formula underestimates HR_max⁡_ for people over 40 years old, while other formulas overestimate this value or give a more accuracy value for specific ages only. This Haskell's formula feature gives a more conservative approach for the calculation of HR_max⁡_, since it is more suitable for a general use. For this reason, Haskell's formula is the most used in the fitness and medical area. Consequently, following the domain experts' suggestions from the Department of Cardiovascular Risk at UCAM (http://international.ucam.edu/studies/masters-in-cardiovascular-risks/presentation/), we also adopt Haskell's formula in this work.

By means of the parameters described above and thumb tables such as Table 2, we can establish general rules about the level of a physical effort which can be resized if the physical capacity is increased or decreased, as the heart rate to the same intensity of effort decreases or increases [27]. These changes may happen depending on the age, number and frequency of exercise, diseases, and so forth. The next section describes how these rules are defined and used in our approach.

## 5. A Simple Health Care Model to Detect Alert Situations

This section explains the components of our model responsible for representing and reasoning about possible alert situations. Following the mainstream in representing context in information systems (see Section 7), we define an ontology named SHCOntology—simple health care (SHC)—to represent our health care domain (see Section 5.1). Based on this ontology we are able to express expert rules to capture alert situations with different levels of confidence according to the available knowledge (see Section 5.2). Finally, we show how the set of general expert rules can be extended to offer personal recommendations to the users (see Section 5.3).

### 5.1. SHCOntology Representation

**Figure 2:**
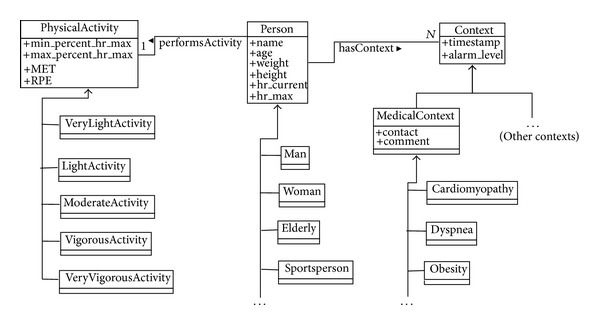
Partial representation of SHCOntology. The main concepts of the ontology are person, physical activity, and medical context.

SCHOntology contains the most relevant terms, relationships, and restrictions in the health care domain proposed in this paper as studied in Section 4. In particular, it contains basic biometrics features, user's contexts (with special interest in a medical context), and different types of physical activities.  Figure 2 offers a schematic and partial representation of our ontology.

In the first place, the ontology captures several biometrics features about each person, including age, weight, height, and current heart rate, among others. The maximal heart rate, which is estimated by means of one of the formulas explained in Section 4 (i.e., the Haskell, Tanaka, or Gellish formulas), is also included. Moreover, each person could be classified according to her gender, physical condition, and so forth. (e.g., a disjoint classification between man and woman is included in the ontology, but they are not exclusive with elderly or sportsperson. Therefore, a person could be classified as an elderly woman who practices sports). The subclasses of person are based on the considered alert situations, but they could be extended taking into account other types of person when necessary.

**Figure 3:**
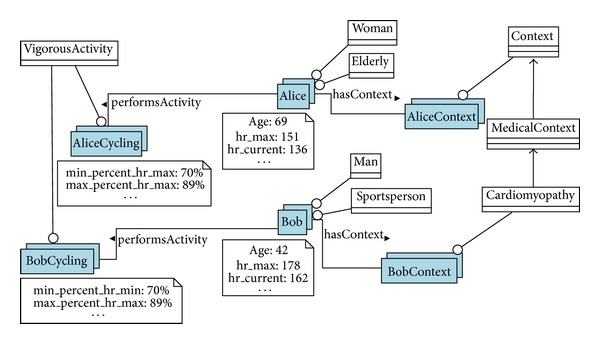
Instantiation of the main concepts of SHCOntology for representing two persons performing cycling: Alice, an elderly woman without any specific medical context, and Bob, a male sportsperson affected by a cardiomyopathy.

Regarding physical activities, they are grouped according to their intensity following the classification proposed elsewhere [28]. For each type of activity, the model records the maximal and minimal limits of the relative intensity of the activity (expressed as percents of the maximal heart rate of the person) along with its associated MET and RPE (see Section 4). As a result, each specific activity (e.g., cycling) is created for each person as an instance of its corresponding intensity class (e.g.,  Vigorous) and it is related to that person through the  performsActivity relationship (see Figure 3 for an example of this relationship between  Bob and  BobCycling instances). Moreover, it is possible to define default values for each intensity level (i.e.,   VeryLightActivity,  LightActivity, etc.) that can be inherited for new activities whose specific values are unknown.

Finally, the ontology also considers several possible contexts for each person. In this manner, several types of medical context (or any other type of context) could be used to describe specific person's situations. Specific context for people affected by cardiomyopathy, dyspnea, and/or obesity, among others, could be represented. The general description of each context includes the timestamp in which the context is considered and the level of the alert (e.g., ignore, low, medium, and high) is generated by the system. Moreover, the  MedicalContext concept contains specific attributes to indicate possible recommendations to the user and emergency call numbers.

Instantiation of this ontology comes from the person's biometric information included in the smartphone. This information—age, weight, height, and so forth—is configured in the device by the user when she uses the application for the first time during the initial setup (it may be changed later at the user's discretion). Regarding the current HR value and the activity, they are provided by the smartwatch and the data mining process, respectively, during the first phase of the process explained in Section 2. Finally, expert medical information (expressed by means of rules; see next subsection) is introduced by the system administrator through a GUI (note that the system administrator acts as a mere translator among the medical and application languages). A complete example of instantiation and relationships among these concepts can be seen in Figure 3.

### 5.2. Defining Expert Rules to Detect Alerts

Once the ontology has been defined, we can use it to reason about the possible alert situations. To do this, we define a set of expert rules using SHCOntology as vocabulary. These rules have been defined and validated in conjunction with the Department of Cardiovascular Risk at UCAM. In particular, we consider 24 expert rules grouped in three levels according to the type of knowledge used by each of them. The levels range from the less informed Level I to the most informed Level III, as explained below. The types of alerts considered for these rules are shown in Table 3. Each alert is related to an action that is performed by the system after the reasoning process. Hence, low-level alerts are associated with a voice alert indicating risky HR values. Medium-level alerts extend this voice alert with a recommendation (e.g., to stop the activity or to reduce the intensity) based on the person's physical condition, activity, and/or medical context. Finally, high-level alerts generate an automatic emergency call if the person does not cancel the alert in 10 seconds. We explain next an illustrative portion of the set of expert rules shown in Algorithm 1 following the knowledge-based level partition.

Level I deals only with HR data. Additional knowledge such as the activity, physical condition, and medical context is considered as unknown at this level. Rule *R*_1_ in Algorithm 1 is an example of rules at Level I. This rule reads “if the person's HR exceeds her estimated maximal HR, then set the alarm level to value* High* for this context.” Observe that  exceeds and  set are functions, where the former checks if the first value is higher than the second one and the latter sets the value of a given attribute.

Level II includes knowledge of the person's condition, specific activity type, or a combination of both. Thus, rule *R*_7_ is an example of including person's condition knowledge. It considers the classification of the  Person concept to refine the basic rules at Level I. In this case, knowing that the person is athletic, exceeding the estimated maximal HR reduces the alert level to* Low*, since it is possible that she is currently practicing some sport. On the other hand, rule *R*_11_ shows an example of a rule taking into account activity knowledge. Now the relative intensity of the activity amends the estimated maximal HR when checking if the current HR exceeds it. Moreover, if the activity is considered as* Very Light* like in rule *R*_11_, the alert level is set to* Medium*. Finally, rule *R*_12_ shows a combination of both types of knowledge. In particular, it sets a* High*-level alert when an elderly person is practicing a vigorous activity and her HR surpasses the maximal value estimated for such an activity.

Rules at Level III include knowledge of the person's medical context. Hence, the classification of the  MedicalContext concept in SCHOntology is employed to refine rules at previous levels. For example, rule *R*_18_ states that if the person has a cardiomyopathy and her HR surpasses the maximal HR recommended for an activity—regardless of its intensity level—then the alert level is set to* High*. Note that this rule refines rule *R*_11_, rising the alert level from* Medium* even though the activity intensity is very light.

It is worth mentioning that during the reasoning process several rules could be activated at different or same levels; that is, conflicts among rules must be taken into account. In case of multiple activations of rules at different levels, the system keeps the highest-level rules and discards the rest. This is due to the fact that the system considers these rules better informed than lower-level ones; that is, they contain more relevant information. In case of multiple activations of rules at the same level, the system keeps rules with the highest alert level and discards the rest. This is due to the fact that the system follows a conservative approach when several types of alerts arise at the same level. Section 6 shows a running example where several rules are activated for the same person and how these rule conflicts are solved. Note also that the current set of rules does not include RPE and MET information yet, but we keep this information in the ontology for future steps. Next subsection explores how to personalize expert rules for specific activities and users.

### 5.3. Extending Expert Rules

Expert rules defined in the previous section follow a general and impersonal approach; that is, they are oriented to capture the most common heart health problems found in literature. However, there are situations where a more personalized approach is necessary, for example, when monitoring a professional sportsperson or elderly people who wish to live independently in their own homes. For these cases it is possible to define customized rules without changing the SHCOntology. Our solution resides in adding new rules with explicit references to the person or activity of interest. The main goal of this kind of rules is to offer personalized recommendations to the users more than detecting high-risk situations. Therefore, the majority of these extended rules set the alert level to* Medium* and include a personalized recommendation.

Algorithm 2 shows two personalized expert rules. *R*_*P*1_ is addressed to professional cyclists during their training sessions. If their HR exceeds a constant value, a recommendation is issued to reduce speed during a period of time. On the other hand, *R*_*P*2_ aims to offer a recommendation to elderly people when climbing stairs too fast. Note that both rules state explicitly the activity to be monitored and the maximal HR threshold instead of using variables. Moreover, *R*_*P*1_ uses an explicit instance of context as well. Observe also that the consequent in the rules contains personalized recommendations.

As personalized rules would be better defined by physicians, telecarers, or even the users themselves, a friendly interface for them should be provided in future versions of the system. In the current version expert rules (personalized or not) are defined using Jena (http://jena.apache.org/) by ontology experts. Jena is a Java framework for building Semantic Web applications which also contains a RETE rule-based reasoner with its own rule language.

## 6. Running Scenario

This section shows how the ontology and rules presented in Section 5 could be used for representing and reasoning about possible alert situations for two different persons.

Figure 3 offers a partial representation of the information captured in SCHOntology about two persons, namely,  Alice and  Bob. In order to explain how the rules are used, we consider an illustrative scenario in which they are practicing cycling. More examples of different users have been used to validate the second phase of the system (see Section 2). The outputs of such examples have been successfully validated by the domain experts from the Department of Cardiovascular Risk at UCAM.

Alice is an elderly woman without any specific medical context and Bob is a male sportsperson affected by a cardiomyopathy. Alice is 69 years old; her weight and height are 70 kg and 1.65 m, respectively. The maximal heart rate considered for her is hr_max⁡ = 151 (this value is obtained using Haskell's formula; see (4)). On the other hand, Bob is 42 years old; his weight and height are 70 kg and 1.82 m, respectively. His maximal heart rate is hr_max⁡ = 178 (estimated again by means of Haskell's formula, (4)).

We suppose that both Alice's and Bob's heart rates increase during cycling, a vigorous activity. The current heart rates registered for Alice and Bob are hr_current = 136 and hr_current = 178, respectively. These data are obtained from the smartwatch and the smartphone they are wearing while practicing the activity.

According to the knowledge captured in the ontology for Alice, rule *R*_12_ is activated (see Algorithm 1). This rule sets a* High*-level alert because Alice is an elderly person performing a vigorous activity and her current heart rate *hr*_*current* exceeds the maximal limits according to her own value of *hr*_*max* and the relative intensity of the activity *max*_*percent*_*hr*_*max*, which is set to 89% (see Figure 3).

On the other hand, two rules are activated when the system reasons about Bob's context: *R*_7_ at Level I and *R*_18_ at Level II (see Algorithm 1). *R*_7_ concludes that the high value of hr_current = 136 is not significant due to the physical condition of Bob; that is, Bob's alarm situation is set as* Low* because he is a sportsperson. *R*_18_ is also activated when a specific Bob's medical context is taken into account. As Bob is affected by a cardiomyopathy, a* High*-level alert is suggested. Consequently, two different alarm situations are inferred using different rules. This conflict must be solved taking into account the rules' level. Hence, the reasoning process sets a* High* alert according to rule *R*_18_ because it belongs to a more informed level than rule *R*_7_ (see Section 5.2).  Table 4 summarizes the alert levels inferred for Alice and Bob.

## 7. Related Work

### 7.1. Physical Activity Recognition

Physical activity is increasingly being more studied with the aim of identifying its intensity and recognizing the activity being performed. Benchmarking on activity recognition task is presented in [29] using a SVM classifier. In another study, the PAMAP2 dataset [6] was recorded on 18 activities with 9 subjects, wearing 3 inertial measurement units and a heart rate monitor. Authors have created a new dataset for physical activity monitoring and it has been made publicly available. In this paper we use this dataset to predict physical activity. It focuses on four classification tasks: intensity estimation, basic activity recognition, background activity recognition, and all activity recognition. In general, very good performance is achieved for all of them, where the best accuracy is given by the K-NN and the boosted decision tree classifiers. Another relevant work by Nam and Park [30] uses a single triaxial accelerometer and a barometric sensor for physical activity recognition. This work is oriented to prevent baby and child accidents such as unintentional injuries at home; however no fine-grained activities are recognized.

With respect to these previous works, our aim is not only to reduce the number of required sensors to recognize activity, but also disguise them in everyday devices such as smartphones and smartwatches. Hence, we remove the ankle sensor and the specific heart sensor attached to a chest band employed in such previous works but keeping similar results. In this manner we obtain a nonintrusive system that can be used in user's daily activities.

It is also worth mentioning that there exist several commercial applications in this direction, specially designed for smartphones and other mobile devices [31–33]. However, although these devices share a nonintrusive philosophy as in our proposal, they do not offer a medical expert rule system to take advantage of the data these devices provide. Moreover, our rules can be adapted to the specific conditions of each subject, which augment the value of our proposal.

### 7.2. Ontologies for Modeling Health Context Information

Ontologies have been used in several context-awareness research domains, including e-health. The definition and use of ontologies in the medical domain represent an active research field, as it has been recognized that ontology-based systems can be used to improve the management of complex health systems [3]. Ontology provides reusable terminology resources for clinical systems and for managing organizational knowledge and cooperative work among care networks. Bettini et al. [34] show a set of requirements that context modeling and reasoning techniques should meet based on database modeling techniques and on ontology-based frameworks for knowledge representation.

However, despite increasing interest in the idea of smart homes as part of an integral health care system, there are few researches about how to cope with the context modeling in this direction. Lee and Kwan [35] define a platform for a smart home health care system, but their context model focuses on social relationships between users and medical experts, in a different line from our work. Hristoskova et al. [36] develop an ambient intelligence framework which provides a dynamic adaptation of prebuilt medical workflows taking into account the clinician's location in order to ensure in time intervention in case of an emergency. The proposed framework is able to change context at runtime in case new services are registered, new rules are defined, or failure/overload of the network occurs. However, the proposed context model is more focused on medical actuation. Muñoz et al. [37] present a decision support system to help caretakers when an alarm is raised in a smart home. Caretakers are presented with graphic and textual information and simulation software to analyze the possible alerts. In this work, ontologies are adopted to represent the smart home structure and along with the inhabitant's biometric data. However, only three simple activities are considered: sleeping, resting, and active.

In this paper we have developed a context-aware model that provides biometric information, a classification of activities, and information about the user's medical context. In this manner, we are able to define expert rules that include all this knowledge to capture possible alert situation and prevent risk cardiac problems.

## 8. Conclusion and Future Work

This paper has presented a novel rule-based system for heart health risk assessment using sensors embedded in a smartphone and a smartwatch. The system provides a nonintrusive solution by using the data from sensors in both devices to determine the physical activity performed by the user. Then, this activity information is combined with the user's heart rate obtained from the smartwatch and other biometrics features to infer possible alert situations and suggestions oriented to prevent cardiac risk.

Regarding the main contributions of this paper, on the one hand our approach is able to determine everyday activities by means of data mining techniques. We have validated, using real data, that it is possible to set the specific activity with accuracy up to 90% employing only inertial sensors from a smartphone and a smartwatch. On the other hand, this work presents ontology as a context model for representing the health knowledge in our system, including fitness status, biometric data, and medical information related to the user and relevant information about the physical activities she is doing in every moment. Such information is used together with a personalized set of expert rules to determine if the person could be suffering from a heart problem or even suggest her some recommendations about the exercise being performed. Consequently, the system provides high-accuracy, unobtrusive solution which offers personalized recommendations to the users in order to detect any high-risk situation in their daily lives.

Regarding future works, firstly we are currently implementing the full system in order to validate the proposal with an enough meaningful sample of people (different age, sex, etc.). Furthermore, the use of the MET and REP indicators will be studied with the aim of taking into account the subjective factor of exercise when defining expert rules. This will provide a more personalized set of rules. Another future work includes storing and analyzing historical information about the person to improve our system in several ways. Firstly, some patterns could be discovered in a long term, such as changes according to the seasons or circadian rhythms. Secondly, by using historical data some anomalies could be determined, such as frequent periods of ectopic heartbeat or similar. Finally, another promising use of the historical information is the possibility of making the system capable of adapting to the user. With such an extension the users could send information to the system to indicate whether the level of suggestion or alarm is appropriated or not. This feedback can then be used by the system to adapt the set of rules and handle some changes in the users' habits or fitness in a lifelong learning mode.

## Figures and Tables

**Algorithm 1:**
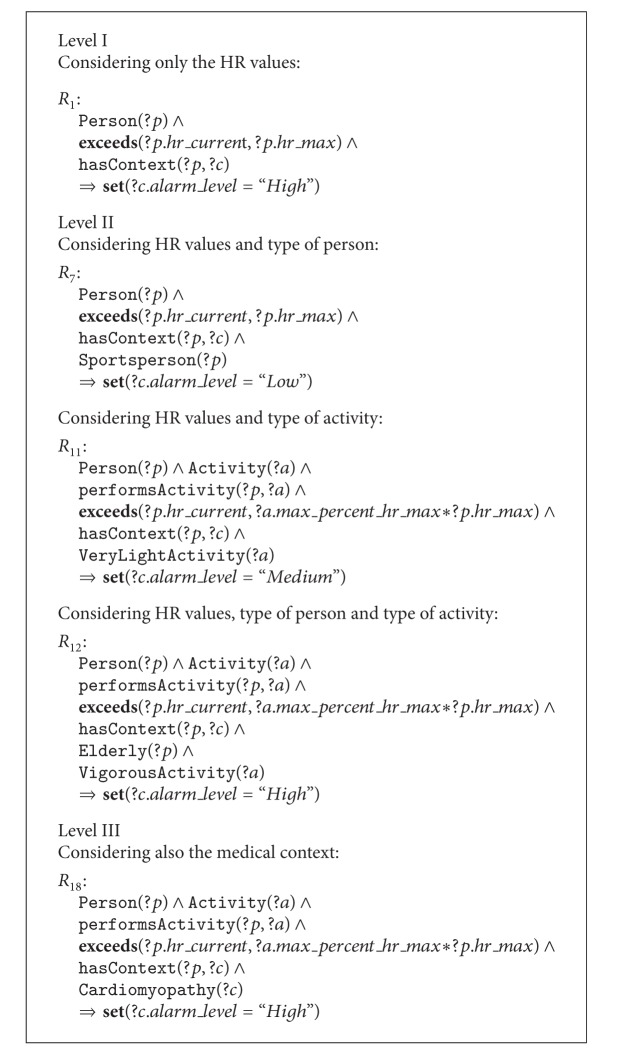
Several examples of general expert rules divided into levels according to the knowledge contained in them.

**Algorithm 2:**
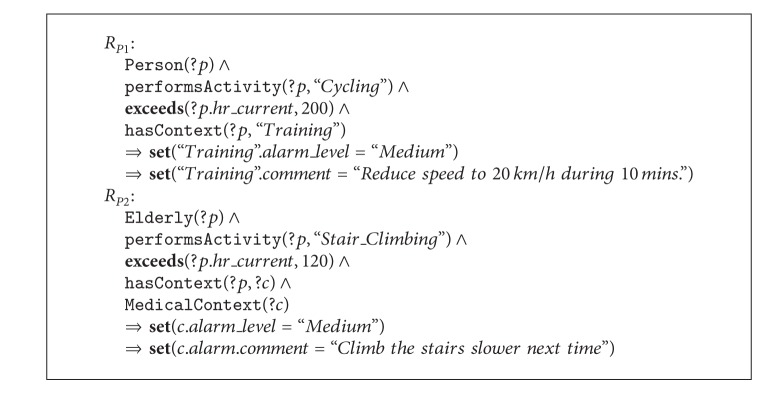
Two examples of personalized expert rules.

**Table 1:** Performance measures.

Full dataset			
Classifier	Precision	*F*-measure	Accuracy
Boosted C4.5	0.9997	0.9994	0.9995
kNN	1.00	1.00	1.00

Reduced dataset			
Classifier	Precision	*F*-measure	Accuracy

Boosted C4.5	0.9968	0.997	0.997
kNN	0.993	0.993	0.993

**Table 2:** Cardiovascular rules table.

	Minimum cardiovascular benefit	Aerobic limit	Anaerobic threshold	Severe exercise
Borg scale RPE	11 (fairly light)	14 (between somewhat hard and hard)	17 (very hard)	18–20 (extremely hard to exhaustion)
% VO_2max⁡_	50%	60–65%	80–85%	≥85%
% HR_max⁡_	70%	75–80%	90–92%	95–100%
Ventilatory responses	Unnoticeable change	Still barely noticeable	Difficult to speak	Exercise hyperpnea, cannot speak

**Table 3:** Alert levels inferred by expert rules and their associated actions.

Alert level	Action
Ignore	N/A
Low	Voice alert
Medium	Voice alert + recommendation
High	Emergency call

**Table 4:** Alert levels inferred by expert rules for two persons performing cycling: Alice, an elderly woman without any specific medical context, and Bob, a male sportsperson affected by a cardiomyopathy.

	Level I	Level II	Level III	Resulting alarm
Alice	—	High (since *R*_12_)	—	High
Bob	—	Low (since *R*_7_)	High (since *R*_18_)	High
